# Assessing 2‑Fluorobutane (CH_3_CHFCH_2_CH_3_) as a Climate-Friendly Alternative: Atmospheric Chemistry and Global Warming Potentials

**DOI:** 10.1021/acsearthspacechem.6c00059

**Published:** 2026-04-06

**Authors:** Elena Jiménez, Laura Gómez-Montes, José Albaladejo, Ole J. Nielsen

**Affiliations:** † Instituto de Investigación en Combustión y Contaminación Atmosférica (ICCA), 16733 Universidad de Castilla-La Mancha. Camino de Moledores, s/n., 13071 Ciudad Real, Spain; ‡ Departamento de Química Física, Universidad de Castilla-La Mancha, Avda. Camilo José Cela, 1B., 13071 Ciudad Real, Spain; § Copenhagen Centre for Atmospheric Research, Department of Chemistry, University of Copenhagen, Universitetsparken 5., 2100 Copenhagen, Denmark

**Keywords:** haloalkanes, hydrofluorocarbons, kinetics, atmospheric lifetime, radiative forcing, global warming, climate change

## Abstract

2-Fluorobutane (HFC-3-10-1se, CH_3_CHFCH_2_CH_3_), a simple fluoroalkane, has received less attention than other widely used hydrofluorocarbons. Understanding its atmospheric chemistry is essential for evaluating its potential as a low- Global Warming Potential (GWP) alternative in industrial applications. Currently, only estimates of GWP relative to carbon dioxide (CO_2_) at time horizons of 20 and 100 years (GWP_20_ and GWP_100_) have been reported to be 4 and 1, respectively, based on the calculated radiative efficiency (RE) and estimated rate coefficients (*k*_OH_) for the gas-phase reaction of hydroxyl (OH) radicals. Here, we present the first experimental kinetic study of the gas-phase reaction of OH radicals with CH_3_CHFCH_2_CH_3_ using a pulsed laser photolysis/laser-induced fluorescence technique (PLP-LIF) (*T* = 264.0–353.2 K; *P* = 60–230 Torr of helium). Contrary to previous estimates, no temperature dependence of the OH-rate coefficients, *k*_OH_(*T*), was observed. A weighted average of *k*_OH_(*T*)=(1.75 ± 0.56)×10^–12^ cm^3^ molecule^–1^ s^–1^ corresponds to a global tropospheric lifetime, τ_OH_, of 6.7 days. Additionally, the ultraviolet (UV, λ = 190–300 nm) and infrared (IR, 3500–500 cm^–1^) absorption cross sections of CH_3_CHFCH_2_CH_3_ were determined at 298 K by gas-phase UV and Fourier Transform infrared (FTIR) spectroscopies. No absorption above 219 nm was observed, therefore UV photolysis of 2-fluorobutane in the solar actinic region (λ > 290 nm) is not expected. From the IR absorption cross sections, the instantaneous radiative efficiency (RE_inst_) was calculated to be 0.0562 W m^–2^ ppbv^–1^, while RE with atmospheric lifetime correction and adjustment for stratospheric temperature was 0.0037 W m^–2^ ppbv^–1^. Using lifetime corrected RE and τ_OH_ values, GWP_20_ and GWP_100_ were calculated to be 0.220 and 0.062. These findings suggest that its rapid atmospheric removal minimizes its contribution to climate change.

## Graphical abstract

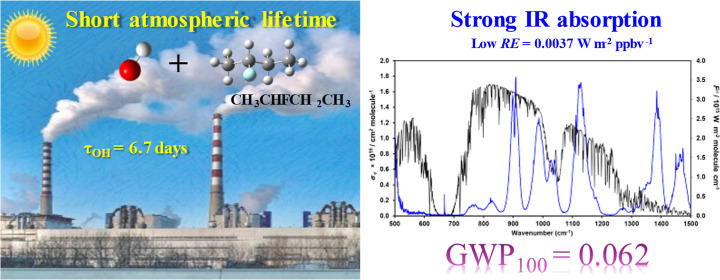

## Introduction

Haloalkanes, which include chlorofluorocarbons (CFCs), hydrochlorofluorocarbons (HCFCs), and hydrofluorocarbons (HFCs), have been widely used in many different applications, such as refrigerants, solvents, and propellants. These compounds play a significant role in atmospheric chemistry due to their diverse environmental impacts, particularly their contribution to radiative forcing and stratospheric ozone depletion. As HFCs do not contain chlorine (Cl) atoms, they do not contribute to destroying stratospheric ozone. However, new commercial refrigerators and freezers containing HFCs with a global warming potential relative to carbon dioxide (CO_2_) at a horizon time of 100 years (GWP_100_) greater than 2500 were banned since 2020 under strict controls on the European Union (EU) Regulation No. 517/2014,^1^ which was adopted to reverse the increase in fluorinated greenhouse gas emissions. That threshold for considering HFC as a high-GWP species was reduced in 2022 to GWP_100_ > 150, as indicated in the EU regulation no. 2024/573.^2^

The simple fluoroalkane, 2-fluorobutane, CH_3_CHFCH_2_CH_3_, has received less attention compared to more extensively used HFCs. While 2-fluorobutane is not currently used and monitored in the atmosphere, the increasing EU regulations on high-GWP HFCs leads to a greater focus on alternative low-GWP fluorinated compounds.^2^ Without accurate measurements of the atmospheric lifetime (τ) and radiative efficiency (RE) of 2-fluorobutane, it would be difficult to predict its impact on changes in Earth’s energy balance. For that reason, understanding the tropospheric chemistry and the radiative properties of 2-fluorobutane is essential for evaluating its potential as a low-GWP alternative before use in industrial applications.^2^

The atmospheric fate of fluoroalkanes is primarily governed by their reaction with hydroxyl (OH) radicals. Thus, knowing the rate coefficient, *k*_OH_(T), for the gas-phase reaction of OH with CH_3_CHFCH_2_CH_3_ (reaction R1) is essential to estimate the atmospheric lifetime due to the OH-reaction (τ_ΟΗ_) which directly impacts GWP.

R1 $$OH+{CH}_{3}{CHFCH}_{2}{CH}_{3}\to Products$$

To our knowledge, no experimental study on the OH-reactivity of CH_3_CHFCH_2_CH_3_ has been reported in the literature. Only an estimate of *k*_OH_(T) and τ_OH_ relative to that of methyl chloroform (CH_3_CCl_3_) at a mean tropospheric temperature of 272 K was reported by Burkholder et al.^3^ *k*_OH_(272 K) was estimated using the structure–activity relationship (SAR) model.^4^ These authors reported a τ_OH_ for CH_3_CHFCH_2_CH_3_ of 0.1 years, indicating that 2-fluorobutane is expected to be a short-lived species. Burkholder et al.^3^ also performed quantum chemical calculations using the second-order Møller–Plesset perturbation theory with a DZP++ basis set to obtain the infrared (IR) absorption spectrum of CH_3_CHFCH_2_CH_3_. This IR spectrum was then used to calculate the instantaneous radiative efficiency, RE_inst_ (0.048 W m^–2^ ppbv^–1^) and RE with atmospheric lifetime correction and adjustment for accounting for stratospheric temperature effects (0.014 W m^–2^ ppbv^–1^). The reported GWP_20_ and GWP_100_ values for CH_3_CHFCH_2_CH_3_ were 4 and 1, respectively. As stated by Burkholder et al.^3^ “A thorough experimental evaluation of a targeted HFC’s atmospheric lifetime and climate metrics is always highly recommended”. Therefore, we report here the first experimental kinetic study of reaction R1 between 264.0 and 353.2 K. Absolute *k*_OH_(T) values were determined by the pulsed ultraviolet (UV) laser photolysis (PLP)-laser-induced fluorescence (LIF) technique.

As far as we know, the UV photochemistry of fluorobutanes is not known. For fluoropropanes, like CHF_2_CH_2_CF_3_ (HFC-245fa) and CF_3_CHFCF_3_ (HFC-227ea), only estimates of the absorption cross section at the Lyman-α wavelength (121.567 nm) have been reported.^5,6^ These estimates assume that the absorption cross section is the same as that of CHF_3_ (HFC-23). Although 2-fluorobutane is not expected to absorb UV radiation in the solar actinic region (λ > 290 nm), in this work we measured the UV absorption spectrum between 190 and 300 nm, to characterize its absorption at the photolysis wavelength, 248 nm, during the kinetic experiments.

The vibrational spectroscopy of 2-fluorobutane has been investigated by several authors. Crowder and Koger,^7^ and Durig et al.^8^ recorded the IR spectrum of 2-fluorobutane in the gas phase between 450 and 1500 cm^–1^ and 50 and 3500 cm^–1^, respectively. These studies focus only on the assignment of the IR bands and not on the determination of the IR absorption cross sections. Only the computed IR spectrum by Burkholder et al.^3^ reported theoretical IR absorption cross sections between 500 and 3500 cm^–1^ at 298 K. Therefore, we report here the first experimental IR absorption cross sections of CH_3_CHFCH_2_CH_3_ between 500 and 3500 cm^–1^ at 298 K by Fourier Transform IR (FTIR) spectroscopy.

## Experimental Methods

### Absolute OH-Kinetics in the Gas-Phase

The experimental system based on the PLP-LIF technique has been widely used in our research group to study the gas-phase kinetics of OH reactions with halogenated species.^9–11^ Briefly, the OH radicals were generated in situ in a jacketed Pyrex reactor (*V* ∼ 200 cm^3^) by UV photolysis of a suitable OH-precursor at 248 nm, radiation from a KrF excimer laser (Coherent, mod. ExciStar 200). At room temperature and above, hydrogen peroxide (H_2_O_2_) was used as an OH precursor, while nitric acid (HNO_3_) was employed at *T* ≤ 285 K. After generating the OH radicals, they were excited at 282 nm (A^2^Σ^+^, ν′ = 1 ← X^2^Π, ν″ = 0) by the second harmonic of a rhodamine-6G dye laser (LiopTech, mod. LiopStar) pumped by the second harmonic of an Nd/YAG laser (InnoLas, model SpitLight 1200). The repetition rate of both lasers was set to 10 Hz. The laser-induced fluorescence signal (*I*_LIF_) from excited OH was detected around 308 nm by a photomultiplier tube and then transferred to a personal computer for further analysis. *I*_LIF_ was monitored as a function of the reaction time in both the presence and absence of 2-fluorobutane.

### Introduction of Gases and Measurements of CH_3_CHFCH_2_CH_3_ Concentration

Gaseous HNO_3_ and H_2_O_2_ were introduced in the reactor by bubbling helium (99.999%, Nippon Gases) through an aqueous solution of the OH-precursor (HNO_3_ 65% w/w and H_2_O_2_ > 50% v/v from Scharlab). The aqueous solution of H_2_O_2_ was preconcentrated as described in Albaladejo et al.^12^ Gaseous mixtures of CH_3_CHFCH_2_CH_3_ in helium were prepared in a 10 L storage bulb by introducing a partial pressure of 2-fluorobutane (*p*_HFC_) from a gaseous sample (98%, abcr GmbH). The dilution factor of 2-fluorobutane (*f*), defined as *p*_HFC_/*p*_T_ where *p*_T_ is the total pressure inside the storage bulb (*p*_T_ = *p*_HFC_ + *p*_He_, usually 760 Torr), ranged from 2.13 × 10^–3^ to 6.97 × 10^–2^, as shown in Table S1 of the Supporting Information.

All gases were introduced into the reactor using calibrated mass flow controllers (MFCs). Especially important is the calibration of the mass flow rate of 2-fluorobutane/He mixtures (*F*_R_). Examples of the calibration are shown in Figure S1 in the electronic Supporting Information. For the highest dilution factor employed (*f* = 6.97 × 10^–2^), the calculated *F*_R_ from off-line measurements of the pressure increase in a known volume over time was 61% of the *F*_R_ value set in the MFC. This difference is reduced for lower dilution factors and, e.g., calculated *F*_R_ was 92% of *F*_R_ set for *f* = 2.13 × 10^–3^. The mass flow rates set in the experiments at the investigated temperatures for diluted 2-fluorobutane, OH-precursor/He (*F*_Prec_), and He (*F*_He_) are summarized in Table S1. As shown in this table, at constant temperature and total pressure (P_T_), [CH_3_CHFCH_2_CH_3_] in the reactor was varied by changing *F*_R_ ((0.11–7.62) × 10^15^ molecules cm^–3^). The total mass flow rate through the reactor was kept constant to avoid any change in the OH precursor concentration that affected the reactivity in the absence of 2-fluorobutane.

### Kinetic Data Analysis

Under pseudo-first order conditions (i.e., 2-fluorobutane in large excess with respect to OH radicals), *I*_LIF_ follows a single exponential function. Some examples of the linearized temporal evolution plots of *I*_LIF_ at several temperatures are shown in Figure S2. For a given concentration, [CH_3_CHFCH_2_CH_3_], and temperature, the pseudo-first order coefficient (*k*′) is obtained from the slope of the corresponding linearized *I*_LIF_ decay. Under these conditions, there is a linear relationship between *k*′ and [CH_3_CHFCH_2_CH_3_], as shown by eq E1.

E1 $$k^{\prime }=k_{OH}\left(T\right)\left[{CH}_{3}{CHFCH}_{2}{CH}_{3}\right]+k_{0}^{\prime }$$

In the absence of 2-fluorobutane, the loss of OH is due to the reaction with the OH precursor and diffusion out of the detection zone (*k*_0_^′^). The individual bimolecular rate coefficients *k*_OH_(*T*) listed in Table S1 were determined from *k*′ vs [CH_3_CHFCH_2_CH_3_] plots. However, to compare kinetic data recorded under different experimental conditions, such as total pressure or concentration of OH precursor, it is better to correct *k*′ with *k*_0_^′^ according to eq E2. The plot of all *k*′ – *k*_0_′ vs [CH_3_CHFCH_2_CH_3_] is shown in Figure 1.

E2 $$k^{\prime }-k_{0}^{\prime }=k_{OH}\left(T\right)\left[{CH}_{3}{CHFCH}_{2}{CH}_{3}\right]$$

**Figure 1:**
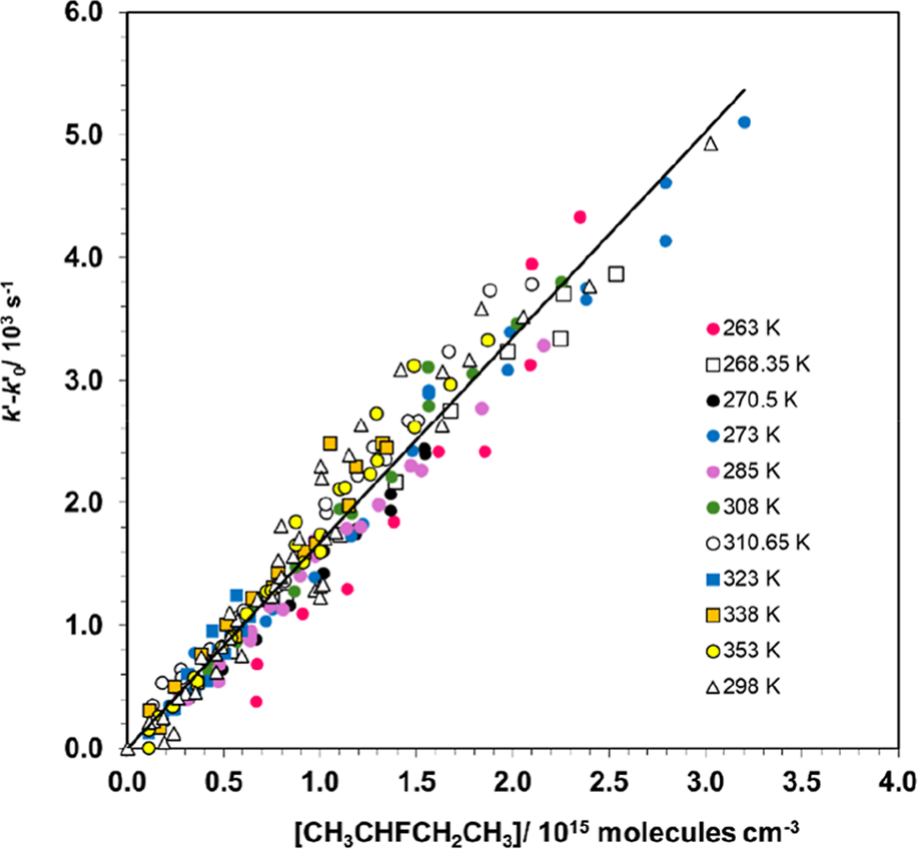
Combination of all kinetic experiments as a function of temperature according to the plot of eq E2. Uncertainties in *k*′ – *k*_0_′ values were not plotted for clarity.

### UV and IR Absorption Spectroscopies in the Gas Phase

The gas-phase UV spectra of 2-fluorobutane were acquired over the 190–300 nm wavelength interval, with a spectral resolution of 3 nm using an f/2 spectrograph (StellarNet, BLACK COMET) equipped with a concave holographic grating (590 grooves) and a 2048-pixel charge-coupled device detector. The experimental system has been described in our previous works.^13–15^ Briefly, a deuterium-tungsten light source (StellarNet, Analytical Instrument Systems, model DT2000) is positioned at the entrance of a jacketed Pyrex absorption cell with an optical path length of 107.15 cm. The transmitted light is focused via an optical fiber onto the spectrograph.

Experiments were performed using pure 2-fluorobutane, previously degassed by several freeze–pump–thaw cycles. A total of 16 spectra, recorded after 16 scans to improve the signal-to-noise ratio, were recorded at pressures inside the absorption cell ranging from 1.48 to 27.42 Torr, corresponding to concentrations at room temperature from 4.80 × 10^16^ to 8.88 × 10^17^ molecules cm^–3^, respectively. The relationship between the absorbance (*A*_λ_) and the HFC concentration follows the general form of Beer–Lambert’s law, as shown in eq E3.

E3 $$A_{\lambda }=\sigma_{\lambda }l\left[{CH}_{3}{CHFCH}_{2}{CH}_{3}\right]$$

where σ_λ_ is the absorption cross section at the wavelength λ, which is obtained from the slope of A_λ_versus [CH_3_CHFCH_2_CH_3_] plots, i.e., from $\sigma_{\lambda }l$ being l = (107.0 ± 0.5) cm. Examples of these plots are depicted in Figure S3 for several wavelengths.

The gas-phase IR absorption spectra of 2-fluorobutane were measured at room temperature using an FTIR spectrometer (Bruker, model Tensor 27). Spectra of both pure and diluted samples were recorded in the spectral range from 500 to 3500 cm^–1^, with a spectral resolution of 0.5 cm^–1^. The experimental setups have been already described in previous works.^15–17^ In this work, two IR absorption cells were used. For pure 2-fluorobutane, measurements were carried out using a single-path stainless-steel absorption cell with an optical path length of 10 cm, while for diluted samples of 2-fluorobutane, a multipath IR absorption cell with a set optical path length of 800 cm was used. For pure 2-fluorobutane, a total of 21 spectra were recorded at pressures inside the absorption cell ranging from 2.031 to 9.757 Torr, corresponding to concentrations from 6.58 × 10^16^ to 3.16 × 10^17^ molecules cm^–3^, respectively. Few IR spectra were recorded using diluted samples at total pressures between 20.1 and 100.5 Torr (2.62 × 10^15^–1.31 × 10^16^ molecules cm^–3^) to verify that the results were consistent independently of the total pressure in the absorption cell. In Figures S4 and S5, examples of the IR spectra of pure and diluted 2-fluorobutane, recorded in the mentioned spectral range, are presented.

Beer–Lambert’s law is expressed, in this case, in terms of the absorbance ( $A_{\mathrm{\nu ̃}}$ ) and the absorption cross section ( $\sigma_{\mathrm{\nu ̃}}$ ) at each wavenumber ν̃, as shown in eq E4

E4 $$A_{\mathrm{\nu ̃}}=\sigma_{\mathrm{\nu ̃}}l\left[{CH}_{3}{CHFCH}_{2}{CH}_{3}\right]$$

being l = 10 or 800 cm. As illustrated in the example of Figure S6, no deviation of linearity was observed over the entire concentration range, confirming that the Beer–Lambert’s law is accomplished under the present experimental conditions.

## Results and Discussion

### Kinetic Results: *k*_OH_(T) between 264.0 and 353.2 K

The individual *k*_OH_(*T*) for the OH reaction of 2-fluorobutane is obtained from plots of eq E1 and are listed in Table 1 between 264.0 and 353.2 K. As can be seen, all *k*_OH_(*T*) are on the order of 10^–12^ cm^3^ molecule^–1^ s^–1^.

**Table 1:** Individual Bimolecular Rate Coefficients (±2σ) for the OH + 2-Fluorobutane Reaction Obtained in This Work

*T*/K	P_T_/Torr	*k*_OH_(*T*)/10^–12^ cm^3^ molecule^–1^ s^–1^
264.0	64.4	1.70 ± 0.37
	136.4	1.73 ± 0.38
268.4	81.2	1.66 ± 0.06
	81.2	1.53 ± 0.09
270.0	68.2	1.55 ± 0.16
	68.2	1.52 ± 0.13
273.0	65.7	1.67 ± 0.18
	137.0	1.54 ± 0.18
284.9	68.5	1.56 ± 0.18
	140.0	1.49 ± 0.05
298.0	58.8	1.87 ± 0.38
	74.4	1.92 ± 0.31
	86.0	1.92 ± 0.34
	122.7	1.76 ± 0.45
	228.9	1.91 ± 0.26
308.2	67.6	1.77 ± 0.18
	142.1	1.73 ± 0.19
310.7	67.4	1.73 ± 0.10
	134.0	1.87 ± 0.15
321.4	83.1	1.72 ± 0.21
	88.5	1.96 ± 0.49
338.2	67.0	1.83 ± 0.16
	135.0	1.78 ± 0.09
353.2	66.7	1.88 ± 0.02
	81.8	1.89 ± 0.15
	135.0	1.89 ± 0.17

As shown in Figure 1, the combined *k* ^′^ – *k*_0_^′^ vs [CH_3_CHFCH_2_CH_3_] plot indicates that *k*_OH_(*T*) is independent of temperature between 264.0 and 353.2 K, within the experimental uncertainties. Therefore, the weighted average of the individual *k*_OH_(T) in that temperature range is (1.75 ± 0.56)×10^–12^ cm^3^ molecule^–1^ s^–1^, which is plotted as a blue line in Figure 2. Quoted errors in *k*_OH_(T), blue shade in Figure 2, are only statistical, ±2σ. The kinetic results obtained in this work indicate that OH radicals react with 2-fluorobutane much faster than the predictions from Burkholder et al.^3^ using the SAR method (*k*_OH_(298 K) = 4.39 × 10^–13^ cm^3^ molecule^–1^ s^–1^).

**Figure 2:**
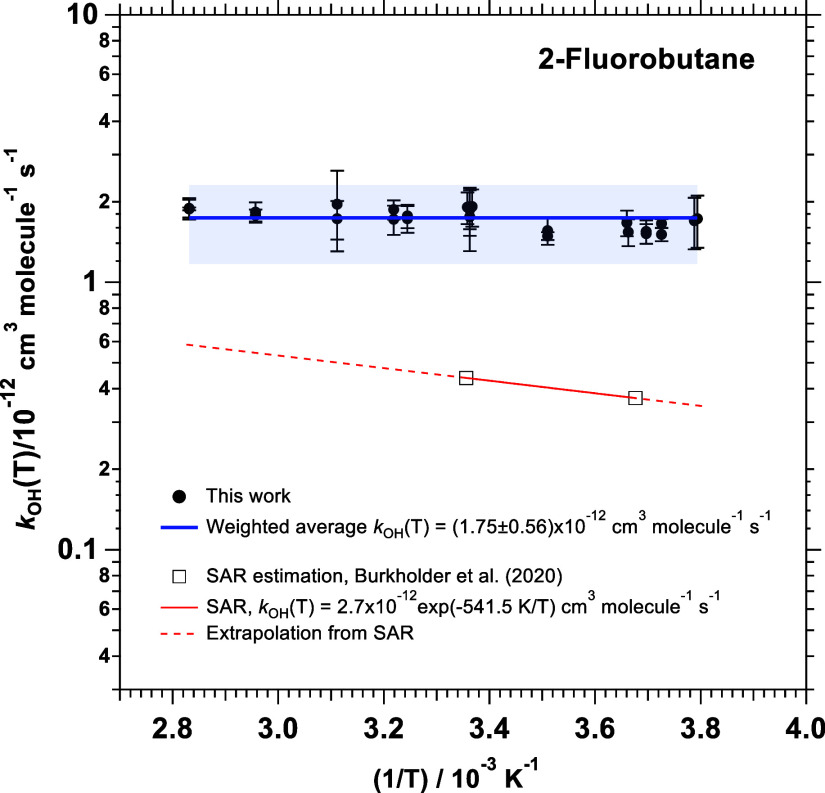
Arrhenius plots of *k*_OH_(*T*) obtained in this work together with estimates from the literature. Quoted errors are statistical ±2σ.

For comparison purposes, *k*_OH_(∼298 K) values for propane and butane and a series of C3- and C4-haloalkanes are summarized in Table S2. When possible, the preferred temperature dependencies of *k*_OH_(T) for these species are those recommended by IUPAC Task Group on Atmospheric Chemical Kinetic Data Evaluation^18^ and JPL-NASA.^19^ As can be seen, the presence of the halogen atom both in all haloalkanes does not impact significantly the OH reactivity, and in general, *k*_OH_(∼298 K) is on the order of 10^–12^ cm^3^ molecule^–1^ s^–1^, except for 2-halopropanes where *k*_OH_(∼298 K) is on the order of 10^–13^ cm^3^ molecule^–1^ s^–1^.

Burkholder et al.^3^ also estimated *k*_OH_(272 K) ∼ 3.6 × 10^–13^ cm^3^ molecule^–1^ s^–1^ that combined with their *k*_OH_(298 K) provides a slightly positive temperature dependence of *k*_OH_(T) with an activation energy (*E*_a_) of around 4.5 kJ/mol (*k*_OH_(T) = *A*exp­{-*E*_a_/*RT*}).

E5 $$k_{OH}\left(T\right)\;∼\;{2.7\times 10}^{-12}\exp\{-542\frac{K}{T}\}{\;cm}^{3}{molecule}^{-1}{\;s}^{-1}$$

The Arrhenius expression given by eq 6 is depicted in Figure 2 as a red line and the extrapolations to 264.0 and 353.2 K are indicated by a dashed red line. Within the uncertainties, no appreciable energy barrier was observed in the present work in the investigated temperature range.

In Table S2, the temperature dependence of *k*_OH_(T) for 2-fluorobutane is compared to that for other halopropanes and halobutanes. Except for the OH + 2-chlorobutane reaction,^20^ the *E*_a_/*R* values are positive ranging from 283 K for OH + 2-bromopropane to 780 K for the OH + 1-iodopropane reaction.^18^ The results reported by Markert and Nielsen^21^ for 1-chloropropane, 2-chloropropane, and 1-chlorobutane are excluded due to large scattering of their data (see Figure S7).

### UV and IR Absorption Cross Sections

Figure 3 presents the UV absorption cross section spectrum of 2-fluorobutane in the 190–300 nm spectral range. As shown, 2-fluorobutane does not appreciably absorb UV radiation above 210 nm. The average σ_λ_ values are listed every 0.5 nm in the Excel file provided in the Supporting Information over the spectral range from 190 to 219 nm.

**Figure 3:**
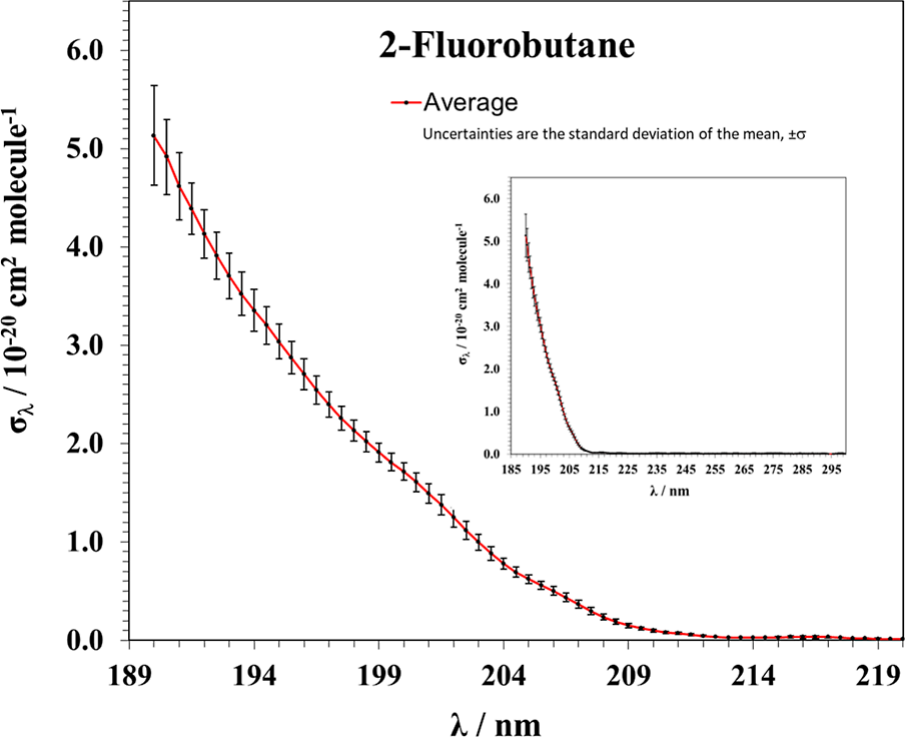
UV absorption cross sections (in base *e*) between 190 and 220 nm for 2-fluorobutane at 298 K. Inset: UV spectrum between 190 and 300 nm.

Figures 4 and S8 present the experimental IR absorption cross section spectrum of 2-fluorobutane in the 500–3500 cm^–1^ range, where absorption features were observed. No significant absorption features are observed between 1550 and 2700 cm^–1^, in agreement with Durig et al.’s observations,^8^ as 2-fluorobutane lacks functional groups absorbing in that spectral region. The average $\sigma_{\mathrm{\nu ̃}}$ values over the spectral range from 500 to 3000 cm^–1^ are listed in the Excel file provided in the Supporting Information every 1 cm^–1^, showing a standard deviation below 5%. This reinforces the consistency of the experimental data and the validity of the applied procedure.

**Figure 4:**
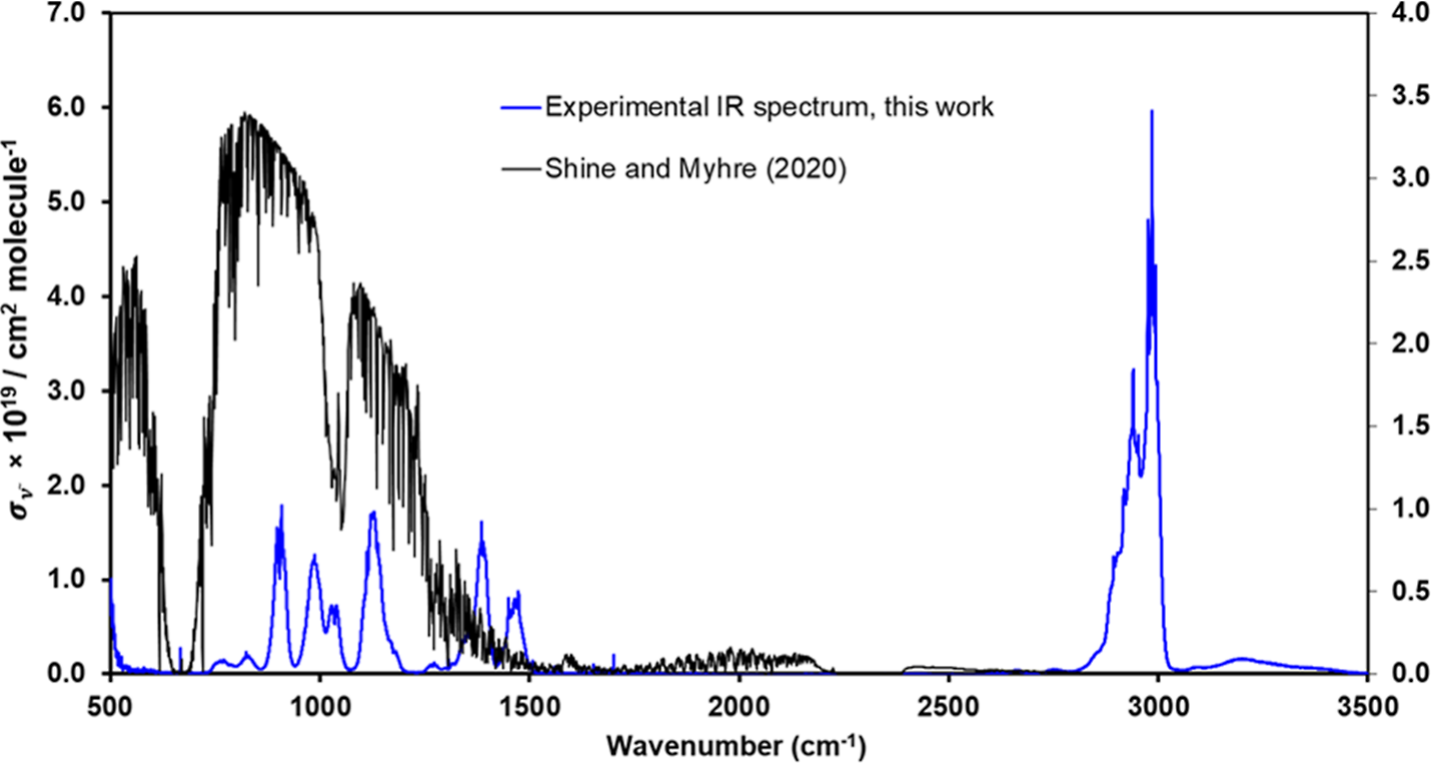
Overlap of the experimental IR absorption spectrum (in base *e*) of 2-fluorobutane at 298 K and *F* ^σ^ from the literature.

The observed IR bands are in agreement with the reported by Durig et al.^8^ at 1 cm^–1^. However, these authors only focused on its spectral analysis and not on the determination of the absolute absorption cross sections. The IR spectrum depicted as a blue dashed spectrum in Figure S8 corresponds to the theoretical data calculated in this study, and the red dashed one corresponds to the theoretical spectrum reported by Burkholder et al.^3^ As stated in the Introduction section, Burkholder et al.^3^ performed quantum chemical calculations using second-order Møller–Plesset perturbation theory with a DZP++ basis set to obtain the IR absorption spectrum of CH_3_CHFCH_2_CH_3_, while in the present study the geometry optimization and frequencies of 2-fluorobutane was computed using Gaussian 16 ^22^ with the CAM-B3LYP/aug-cc-pVTZ.^23–25^ The calculated IR spectrum between 0 and 3500 cm^–1^ was made with a Gaussian peak broadening with 10 cm^–1^ full width at half-maximum. As shown in Figure S8, the computed IR spectra reported here and by Burkholder et al.^3^ are shifted to higher wavenumbers, especially in the C–H bond region (2800–3100 cm^–1^).

### Atmospheric Implications and Climate Metrics

#### Atmospheric Lifetime of 2-Fluorobutane

As stated above, 2-fluorobutane does not absorb UV radiation above 210 nm. Therefore, UV photolysis in the solar actinic region is not a removal pathway in the troposphere. Its reaction with OH radicals is then the main removal process, and τ_OH_ can be considered as the atmospheric lifetime. It is determined using eq 7, where *k*_OH_ is the average value between 264.0 and 353.2 K and [OH]_24h_ = 10^6^ radicals cm^–3^.

E6 $$\tau_{OH}=\frac{1}{k_{OH}{\left[OH\right]}_{24h}}$$

The estimated τ_OH_ is 6.7 days, i.e., ∼0.02 years. The lower *k*_OH_ predicted by Burkholder et al.^3^ translated in a much longer τ_OH_ (0.1 years). The approximate nature of this simple lifetime should be stressed as it assumes a well-mixed distribution, which would be extremely unlikely for CH_3_CHFCH_2_CH_3_. The local lifetime for such a short-lived substance will depend on the time of year and location of its emission. Seasonal variations in the atmospheric OH concentration due to the annual oscillation of the photolytic activity also impacts the instantaneous atmospheric lifetime.

Considering that the time scale of vertical transport from the surface to the stratosphere is generally of weeks to months, depending on location and meteorological conditions, 2-fluorobutane with a lifetime of less than a week is very unlikely to reach the stratosphere. According to Burkholder et al.^3^ calculations the reaction of O­(^1^D) with 2-fluorobutane in the stratosphere is not a competing atmospheric loss process (lifetime of 411 years).

#### Radiative Efficiencies and GWPs

The RE_inst_ for 2-fluorobutane was calculated from the integrated $\sigma_{\mathrm{\nu ̃}}$ for Δν̃ = 1 cm^–1^ in this work and the radiative forcing per integrated absorption cross section (*F* ^σ^ in W m^–2^ cm^–1^ molecule) from Shine and Myhre.^26^ Following the methodology used in our previous works,^15,27^ RE_inst_ was calculated to be 0.0562 W m^–2^ ppbv^–1^, 17.1% higher than the one calculated by Burkholder et al.^3^ (0.048 W m^–2^ ppbv^–1^).

E7 $${RE}_{inst}≅\sum_{500{\;cm}^{-1}}^{1500\;{cm}^{-1}}\sigma_{\mathrm{\nu ̃}}F^{\sigma }\Delta \mathrm{\nu ̃}$$

In Figure 4, the spectral variation of *F* ^σ^ is overlapped with $\sigma_{\mathrm{\nu ̃}}$ . As RE_inst_ refers to how much radiative forcing (i.e., warming effect) a unit mass of 2-fluorobutane causes immediately upon emission, to account for its total climate impact, which depends on how long it stays in the atmosphere, and for stratospheric temperature effects, RE_inst_ was corrected using the atmospheric lifetime due to the reaction with OH. Based on this, the fractional lifetime correction factor (*f*_τ_) for 2-fluorobutane taking into account τ_OH_ = 6.7 days was calculated to be 0.0663. Thus, the resulting corrected RE was 0.0037 W m^–2^ ppbv^–1^, which is about 4 times lower than the reported value by Burkholder et al.^3^ (0.014 W m^–2^ ppbv^–1^).

E8 $$RE=f_{\tau }\times RE_{inst}$$

The estimated lifetime and the lifetime corrected RE were used to derive climate metrics, such as the global warming potential of a gas *i* (GWP_*i*_) relative to CO_2_ using eq 10.^28^

E9 $$GWP_{i}\left(TH\right)=\frac{AGWP_{i}\left(TH\right)}{AGWP_{CO_{2}}\left(TH\right)}$$

AGWP_*i*_ represents the absolute GWP for *i* over a time horizon (TH) and is calculated considering the lifetime corrected RE_*i*_, the atmospheric lifetime τ_*i*_, which is taken as τ_OH_, and the exponential decay of the compound in the atmosphere ([Disp-formula eq11]).

E10 $$AGWP_{i}={RE}_{i}\tau_{i}\left(1-\exp\left(-\frac{TH}{\tau_{i}}\right)\right)$$

AGWP_CO_2__ corresponds to the absolute GWP for CO_2_, which exhibits more complex behavior due to multiple atmospheric removal processes with different time scales. It is expressed as eq 12.

E11 $$AGWP_{CO_{2}}={RE}_{CO_{2}}\int_{0}^{TH}IRF_{CO_{2}}\left(t\right)\;dt={RE}_{CO_{2}}[a_{0}TH+\sum_{i=1}^{3}a_{i}\alpha_{i}(1-\exp\left(-\frac{TH}{\alpha_{i}}\right)]$$

The impulse response function (IRF) describes how emitted CO_2_ decreases in the atmosphere over time, as it is absorbed by the distinct oceanic reservoirs (surface, thermocline, deep ocean) on different time scales. The parameters *a*_i_ and α_i_ used in the integral of the IRF were adopted from Gasser et al.,^29^ with values *a*_0_ = 0.2033, *a*_1_ = 0.3016, *a*_2_ = 0.2836, *a*_3_ = 0.2115, α_1_ = 4.736 years, α_2_ = 34.09 years, and α_3_ = 288.4 years. According to Hodnebrog et al.,^28^ RE_CO_2__ represents the radiative efficiency of CO_2_, with a value of 1.2895 × 10^–5^ W m^–2^ ppbv^–1^, corresponding to a reference concentration of 409.8 ppm of CO_2_. To express AGWP_CO_2__ in terms of kilograms emitted, it is necessary to convert RE_CO_2__ to W m^–2^ kg^–1^. This conversion is performed by multiplying RE_CO_2__ by the factor (*M*_A_/*M*_CO_2__) × (10^9^/*T*_M_), where *M*_A_ is the molar mass of dry air (28.97 g mol^–1^), M_CO_2__ is the molar mass of CO_2_ (44.01 g mol^–1^), and *T*_M_ is the mean dry mass of the atmosphere (5.135 × 10^18^ kg). Using this conversion, RE_CO_2__ is 1.65 × 10^–15^ W m^–2^ kg^–1^. With this value, the AGWP_CO_2__ for time horizons of 20, 100, and 500 years are 2.290 × 10^–14^, 8.064 × 10^–14^, and 2.694 × 10^–13^ W m^–2^ yr (kgCO_2_)^−1^, respectively.

In Table 2, a comparison of GWPs at 20 and 100 yrs obtained in this work and those estimated from Burkholder et al.,^3^ which were also corrected with the estimated lifetime, is presented. As can be seen, GWPs obtained in this work from experimental RE are significantly lower than the theoretical values reported by Burkholder et al.^3^ The difference in the GWP values mainly comes from the shorter atmospheric lifetime of the HFC.

**Table 2:** Comparison of GWPs of 2-Fluorobutane at Horizon Times of 20 and 100 years

TH (yr)	AGWP_CO2_(TH)/W m^–1^ yr (kgCO_2_)^−1^	GWP_TH_	reference
			
20	2.29 × 10^–14^	0.220	This work, experimental
		4	Burkholder et al. (2020), theoretical
100	8.06 × 10^–14^	0.062	This work, experimental
		1	Burkholder et al. (2020), theoretical

In Table 3, a comparison of τ_OH_ and GWP_100_ for 2-fluorobutane is presented for conventional refrigerants such as HFC-134a (R-134a), HFC-152a (R-152a), and newer low-GWP alternatives, such as hydrofluoroolefins HFO-1234yf and HFO-1234ze. Although HFC-134a and HFC-152a are not completely banned yet, both are under global phase-down schedules due to their high GWP. They remain in use in some sectors, but their production and consumption are being strictly reduced under the Kigali Amendment (2016). Thus, R-134a is being replaced by HFO-1234yf in automotive air-conditioning systems, and HFO-1234ze is used as aerosols/foam blowing. The reduction of GWP_100_ in 2-fluorobutane with respect to HFC-134a and HFC-152a is considerable and more than 1 order of magnitude with respect to HFOs. Therefore, in terms of the environmental impact of 2-fluorobutane, our findings suggest that if it were emitted to the atmosphere, its rapid atmospheric removal minimizes its overall contribution to climate change, supporting its consideration as a potential low-GWP alternative in industrial applications.

**Table 3:** Comparison of τ_OH_ and GWPs at Time Horizon of 100 Years for Some HFCs Used as Refrigerants or Propellants

HFC	τ_OH_	GWP_100_	reference
HFC-3-10-1se	6.7 days	0.062	this work
HFC-134a	14 years	1530	AR6 IPCC (2024)^30^
HFC-152a	1.6 years	164	AR6 IPCC (2024)^30^
HFC-1447fz	8 days	0.19	Jiménez et al. (2016)^11^
HFO-1345fz	9 days	0.26	González et al. (2016)^31^
HFO-1234ze (E)	16 days	1.37	Antiñolo et al. (2017)^32^
HFO-1234yf	12 days	0.501	AR6 IPCC (2024)^30^

Based on its physical and chemical properties, 2-fluorobutane seems to be unsuitable as a standalone refrigerant for conventional refrigeration or air-conditioning systems due to its high boiling point (∼25 °C), low vapor pressure (≈53 kPa at 25 °C), and significant flammability. Its use as a foam expansion agent or aerosol propellant is technically feasible, but it would necessitate robust safety measures. Incorporation into refrigerant blends as a minor component could be considered to reduce GWP or tailor thermodynamic properties for specialized applications, provided that flammability and compatibility issues are adequately addressed. For example, its use as a refrigerant or as a component in a refrigerant blend showed that blends of a mixture of HFCs and hydrocarbons like butane, isobutane, and isopentane improve efficiency of refrigeration and air-conditioning systems.^33^ Future research should focus on detailed thermodynamic modeling, life cycle, and safety assessments, necessary before considering large-scale deployment. Additionally, its toxicity for inhalation has also to be evaluated, although based on its structure (similar to other fluorinated alkanes), 2-fluorobutane is likely to be nontoxic.

## Supplementary Material







## Notes

IR absorption cross section (XLSX)
UV-vis absorption cross section (XLSX)
Experimental details, such as experimental conditions , mass flow calibrations, registered OH decays, and IR spectra, examples of the Beer–Lambert’s law plots, detailed comparison of *k*_OH_(T) for 2-fluorobutane with alkanes and other haloalkanes, and experimental IR spectrum with the computed ones (PDF)
