# Supplementary material for: Assessing 2‑Fluorobutane (CH3CHFCH2CH3) as a Climate-Friendly Alternative: Atmospheric Chemistry and Global Warming Potentials
Source: ACS Earth Space Chem. 2026 Apr 6;10(4):1145–52. doi: 10.1021/acsearthspacechem.6c00059 (PMC13094374; doi:10.1021/acsearthspacechem.6c00059)
Supplement: Supplementary file 3 [file sp6c00059_si_003.pdf]

SUPPORTING INFORMATION

Assessing 2-Fluorobutane ($CH_3CHFCH_2CH_3$) as a Climate-Friendly Alternative: Atmospheric Chemistry and Global Warming Potentials

Elena Jiménez,[1,2,*] Laura Gómez-Montes,[1,2] José Albaladejo,[1,2] and Ole J. Nielsen[3]

[1] Instituto de Investigación en Combustión y Contaminación Atmosférica (ICCA). Universidad de Castilla-La Mancha. Camino de Moledores, s/n. 13071 Ciudad Real (Spain).

[2] Departamento de Química Física. Universidad de Castilla-La Mancha. Avda. Camilo José Cela, 1B. 13071 Ciudad Real (Spain).

[3] Copenhagen Centre for Atmospheric Research, Department of Chemistry, University of Copenhagen, Universitetsparken 5, 2100 Copenhagen (Denmark).

Table S1. Experimental conditions, measured *pseudo*-first order rate coefficients (k') and individual bimolecular rate coefficients (±2σ).

T/ K	P_T / Torr	$f \times 10^3$	F_R / sccm	F_{Prec}/ sccm	F_{He} / sccm	[$CH_3CHFCH_2CH_3$] / 10^{15} molecules cm^{-3}	k'_0 / s^{-1}	k'-k'_0 / s^{-1}	$k_{OH}(T)$ / 10^{-12} cm^3 $molecule^{-1}$ s^{-1}
263.8±0.2	64.4	5.44	20-100	30 [a]	380-480	0.67-2.10	807 ± 49	388-3130	1.70 ± 0.37
	136.4	3.87	10-70	30 [a]	330-400	0.36-2.85	625 ± 33	456-4530	1.73 ± 0.38
268.4	81.2	5.44	20-90	40 [a]	390-480	0.53-2.27	218 ± 4	1114-3870	1.66 ± 0.06
	81.2	5.44	30-80	40 [a]	400-480	0.82-1.98	541 ± 22	1237-3240	1.53 ± 0.09
270.1±0.1	68.2	3.87	60-90	30 [a]	390-480	1.02-1.54	303 ± 6	1607-2444	1.55 ± 0.16
	68.2	3.87	20-90	30 [a]	390-460	0.32-1.54	558 ± 22	407-2396	1.52 ± 0.13
273.1±0.1	65.7	5.44	10-80	20 [a]	370-450	0.21-1.98	232 ± 4	354-2507	1.67 ± 0.18
	137.0	3.87	20-80	30 [a]	320-400	0.35-3.20	235 ± 4	496-4820	1.54 ± 0.18
284.9	68.5	3.87	10-90	30 [a]	390-480	0.47-1.47	685 ± 26	550-2303	1.56 ± 0.18
	140.0	3.87	10-70	30 [a]	450-520	0.58-2.16	742 ± 13	954-3285	1.49 ± 0.05
297.4 ± 4.0	58.8	2.13	10-25	10 [b]	260-800	0.86-7.62	665 ± 28	525-1112	1.87 ± 0.38
	74.4	69.7	10-25	5 [a]	1000	1.24-3.31	212 ± 5	1310-5122	1.92 ± 0.31
	86.0	3.31	10-30	10 [b]	970-1000	0.66-2.06	1008 ± 70	881-3746	1.92 ± 0.34
	122.7	4.02	20-100	30 [b]	600-700	0.38-2.06	1796 ± 56	678-3450	1.76 ± 0.45
	228.9	3.86	10-50	30 [a]	350-400	0.53-3.03	1071 ± 49	1102-4446	1.91 ± 0.26
308.2	67.6	3.86	10-100	20 [b]	400-500	0.12-1.46	1009 ± 17	256-2663	1.77 ± 0.18
	142.1	3.93	20-100	30 [b]	600-700	0.42-2.25	889 ± 13	711-3806	1.73 ± 0.19
310.7	67.4	3.93	10-100	10-15 [b]	400-500	0.28-1.49	661 ± 16	646-3152	1.73 ± 0.10
	134.0	3.93	10-100	30 [b]	600-700	0.39-2.10	636 ± 12	738-3788	1.87 ± 0.15
321.4	83.1	2.13	10-100	10 [b]	800-900	0.11-0.59	974 ± 31	132-689	1.72 ± 0.21
	88.5	2.13	10-100	10 [b]	800-900	0.17-0.63	1232 ± 182	233-1248	1.96 ± 0.49
338.2	67.0	3.86	10-100	20 [b]	400-500	0.11-1.32	747 ± 13	315-2485	1.83 ± 0.16
	135.0	3.93	10-100	30 [b]	600-700	0.17-1.15	603 ± 19	170-1978	1.78 ± 0.09
353.2	66.7	3.86	10-100	20 [b]	400-500	0.11-1.26	703 ± 27	8-2234	1.88 ± 0.02
	81.8	2.63	20-100	20 [b]	400-500	0.22-1.14	27 ± 5	1289-2105	1.89 ± 0.15
	135.0	3.86	10-100	20 [b]	600-700	0.16-1.87	453 ± 12	261-3329	1.89 ± 0.17

[a] HNO_3 as OH precursor; [b] H_2O_2/H_2O as OH-precursor.

Calibration of mass flow rates for 2-fluorobutane

Although the mixing ratio (f) of 2-fluorobutane (hereafter R) in the storage bulb ($(2.13\text{-}69.7)\times10^{-3}$) is not as higher as those employed for other halogenated species that present lower OH-reactivity, e.g. halogenated anesthetics, the mass flow controller (MFC) used to introduce diluted 2-fluorobutane in the reactor was calibrated for each f value. The calibration consisted in measuring the increase in pressure (ΔP) over a period of time (Δt) in a known volume (V_f) at room temperature as a function of the mass flow rate set ($F_{R,set}$ = 10-100 sccm, standard cubic centimeter per minute). The calculated mass flow rate ($F_{R,calc}$) according to equation (ES1) is plotted against $F_{R,set}$, as shown in Figure S1.

$$F_{R,\,calc}(\text{sccm}) = V_f(cm^3)\,\frac{273\,K}{T(K)}\,\frac{\Delta P(Torr)}{760Torr\,\Delta t(min)} \quad \text{(ES1)}$$

As shown in Figure S1, the slope of $F_{R,calc}$ versus $F_{R,set}$ plots differs from unity when increasing f due to a change in the gas mixture properties (density, thermal conductivity and heat capacity) with respect to the gas used in the factory calibration (in this case, helium). The highest mixing ratio of 2-fluorobutane corresponds to the first experiments where the estimated rate coefficient by Burkholder *et al.* was assumed. In that case, the slope of the $F_{R,calc}$ versus $F_{R,set}$ plot is 0.61, i.e., the real mass flow rate of 2-fluorobutane/He mixture is 39% lower than that set in the MFC. This manifests the importance of calibrating the MFCs. After confirming that the OH-rate coefficient was faster than the predictions the mixing factors were lowered. However, even at the lowest mixing ratio used in the present study, the slope of the $F_{R,calc}$ versus $F_{R,set}$ plot is 0.92, i.e., the flow rate of 2-fluorobutane/He mixture is 8% lower than that set in the MFC.

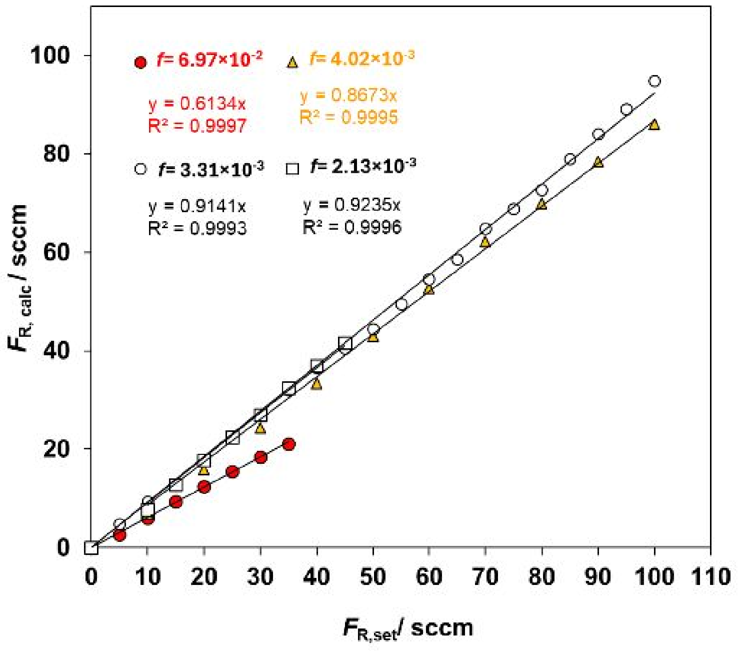

Figure S1. Example of the calibration of the mass flow controller for the mixtures of 2-fluorobutane in helium.

Decays of the I_{LIF} signal

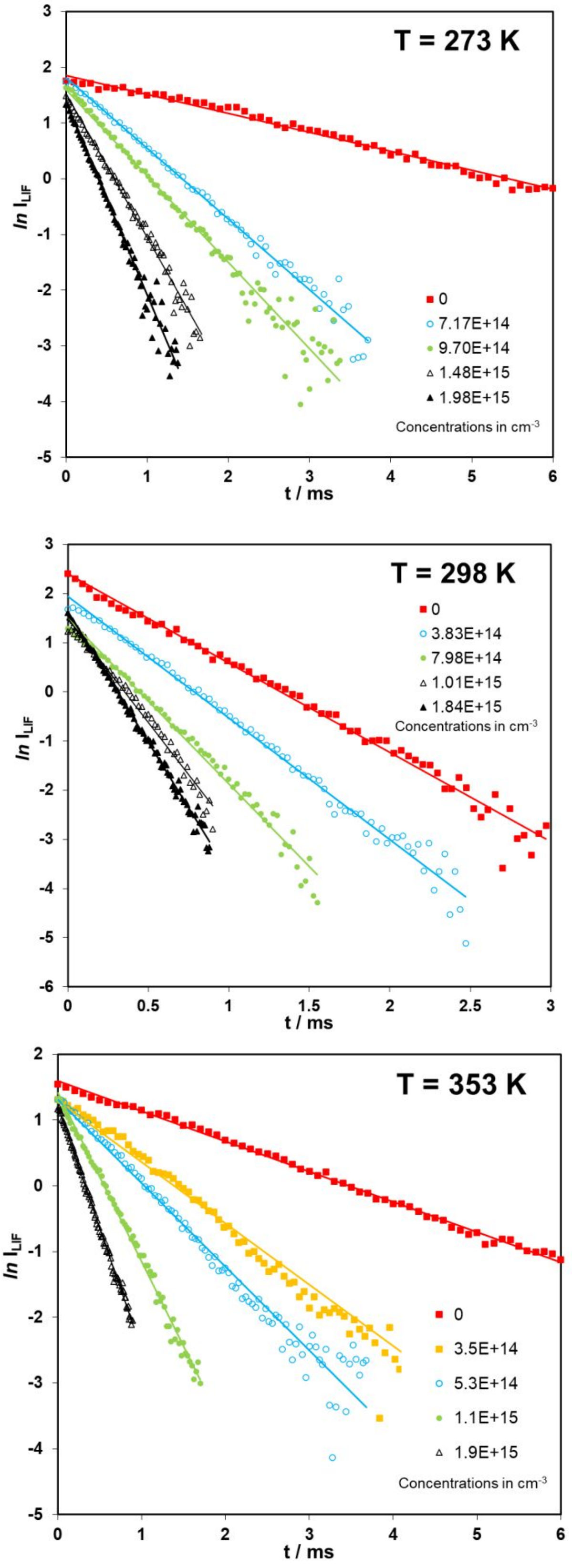

Figure S2. Examples of the LIF decays (linearized) recorded as a function of 2-fluorobutane concentration at several temperatures.

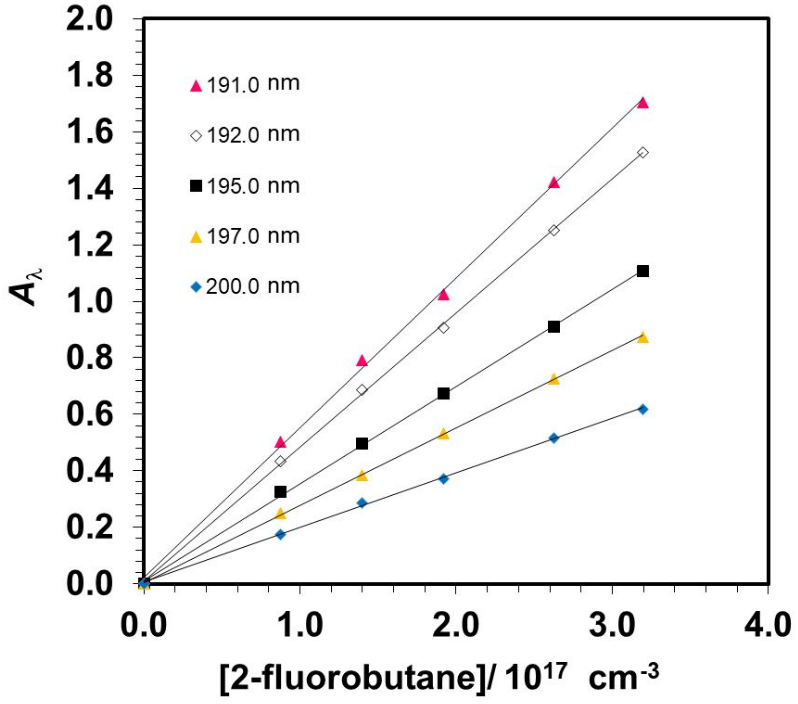

Figure S3. Examples of the Beer-Lambert's law plots for 2-fluorobutane in the UV region.

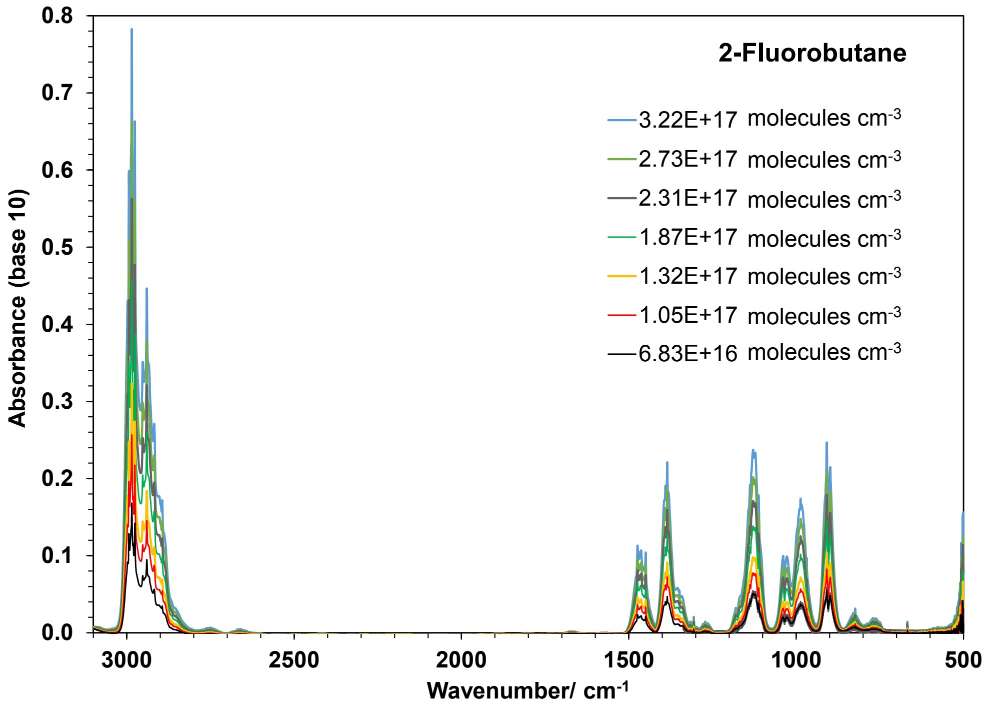

Figure S4. IR spectrum of pure 2-fluorobutane at several pressures.

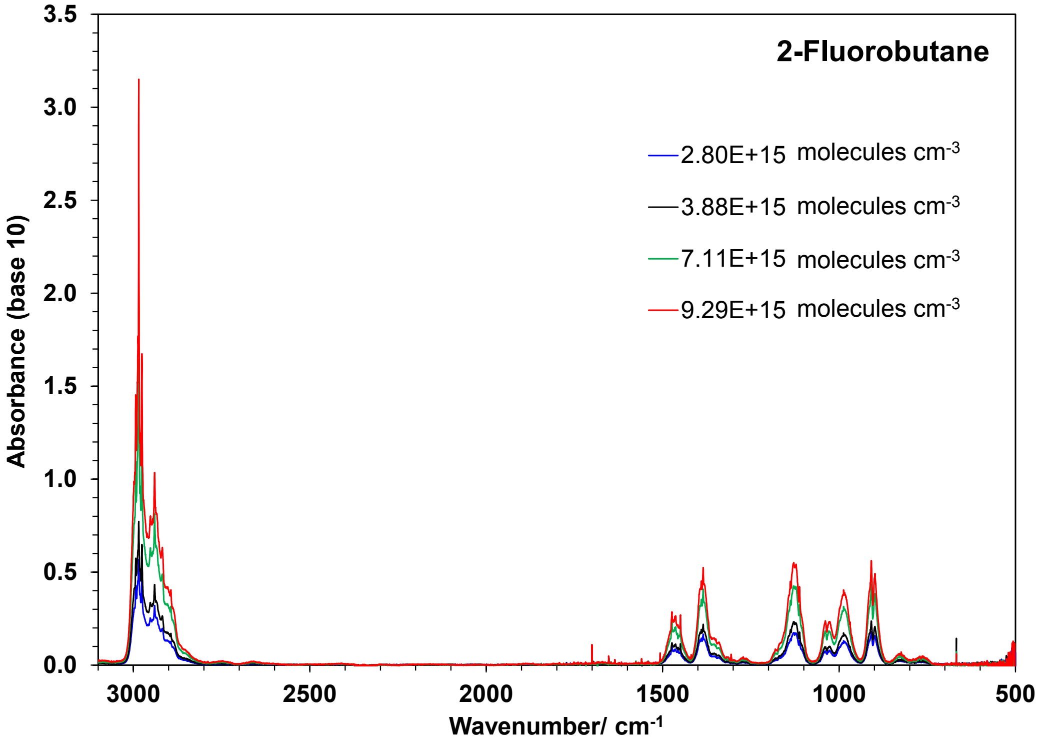

Figure S5. IR spectrum of 2-flurobutane from diluted samples (f=4.02×10^{-3}) at several total pressures (21.5 Torr (blue), 29.8 Torr (black), 54.6 Torr (green) and 71.3 Torr (red)).

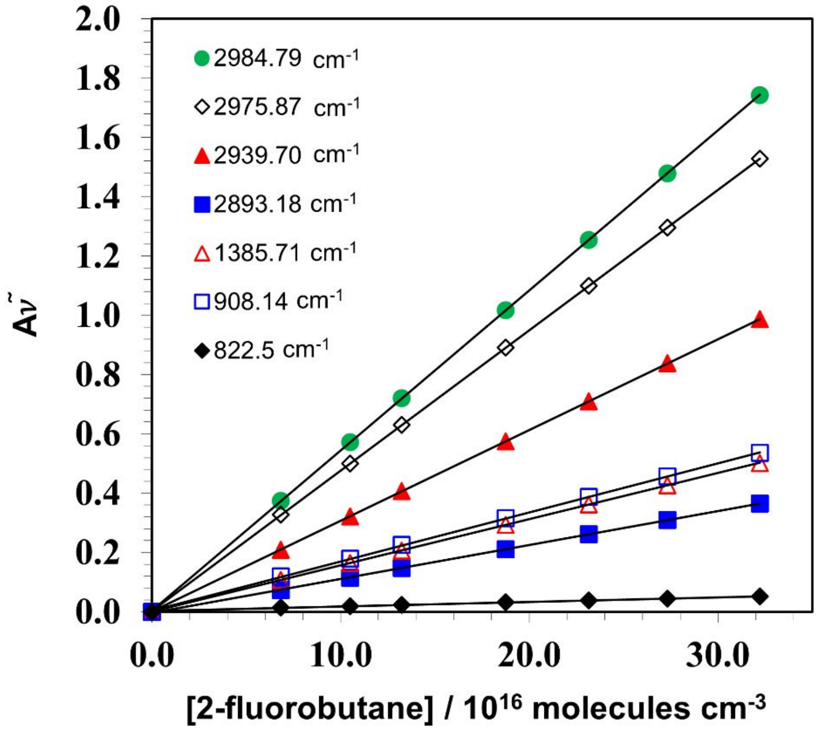

Figure S6. Verification of the Beer-Lambert law for pure 2-fluorobutane at selected wavenumbers.

Comparison of k_{OH}(T) for 2-fluorobutane with alkanes and other haloalkanes.

In summary, the general OH-reactivity trends at room temperature are:

$$k_{OH}(\text{1-halopropanes}) > k_{OH}(\text{2-halopropanes})$$

$$k_{OH}(\text{1-halobutanes}) \cong k_{OH}(\text{2-halobutanes})$$

$$k_{OH}(\text{1-halopropanes}) < k_{OH}(\text{1-halobutanes})$$

$$k_{OH}(\text{2-halopropanes}) < k_{OH}(\text{2-halobutanes})$$

As shown in Table S2, the temperature dependence of k_{OH}(T) has been investigated for 1-halopropanes, except for 1-fluoropropane, and for 2-halopropanes, except for 2-iodopropane. For C3-haloalkanes, E_a/R values are slightly positive for OH-reactions, excluding the values reported by Markert and Nielsen[1] which exhibit large scattering (see Figure S6). The E_a/R values range from 365 K (for the OH+2-chloropropane reaction) to 780 K (for the OH+1-iodopropane reaction). For C4-haloalkanes, the Arrhenius expressions of k_{OH}(T) has only been reported for the OH+1-chlorobutane and 2-chlorobutane reactions. The magnitude of E_a/R values is different and Markert and Nielsen[1] reported a large positive E_a/R for the OH+1-chlorobutane reaction, while Kar and Rajakumar[2] reported a negative E_a/R for the OH+2-chlorobutane reaction. There are still some unknowns on the activation energy for most of the reactions listed in Table 2 and additional kinetic measurements are needed in order to update JPL-NASA and IUPAC evaluations.

Table S2. Comparison of the temperature dependence of k_{OH}(T) and k_{OH} at room temperature (295-303 K) for halopropanes and halobutanes.[a]

	**Species**	***T* range (K)**	**k_{OH}(~298 K)/ 10^{-12} cm^3 molecule^{-1} s^{-1}**	**$A/10^{-12}$ cm^3 molecule^{-1} s^{-1}**	**E_a/R (K)**	**Reference**
***C*$_3$**	propane	190–908	1.11	9.19	630	JPL-NASA, Burkholder et al. (2019)
		200-300	1.10	7.60	585	Atkinson et al. (2006)
	1-fluoropropane	*n.a.*	*n.a.*	*n.a.*	*n.a.*	
	1-chloropropane	253-372	0.98 ± 0.10	5.7 ± 1.0	526 ± 53	Yujing and Mellouki (2001)
		303	1.12 ± 0.28	*n.d.*	*n.d.*	Donaghy et al. (1993)
		295-353	0.96 ± 0.04	100 ± 30	1443 ± 476	Markert and Nielsen (1992)
	1-bromopropane	210-300	1.0	3.91	399	IUPAC 2023
	1-iodopropane	290-380	1.36	18.6	780	IUPAC 2023
	2-fluoropropane	288–394	0.587	3.00	490	JPL-NASA, Burkholder et al. (2019)
	2-chloropropane	233-372	0.70 ± 0.07	2.4 ± 0.6	365 ± 66	Yujing and Mellouki (2001)
		303	0.92 ± 0.23	n.d.	n.d.	Donaghy et al. (1993)
		295-353	0.43 ± 0.04	50 ± 6	1323 ± 847	Markert and Nielsen (1992)
	2-bromopropane	210-335	0.758	1.96	283	IUPAC 2023
	2-iodopropane	298	1.2	*n.a.*	*n.a.*	IUPAC 2023
***C*$_4$**	butane	180-300	2.35	9.8	425	IUPAC 2021, Mellouki et al. (2021)
		185–509	2.40	10.2	430	JPL-NASA, Burkholder et al. (2019)
	1-fluorobutane	*n.a.*	*n.a.*	*n.a.*	*n.a.*	
	1-chlorobutane	298	2.00 ± 0.15	n.d.	n.d.	Loison et al. (1998)
		295-353	1.69 ± 0.09	28 ± 9	842 ± 481	Markert and Nielsen (1992)
	1-bromobutane	298	2.3	*n.a.*	*n.a.*	IUPAC 2023
	1-iodobutane	*n.a.*	*n.a.*	*n.a.*	*n.a.*	
	2-fluorobutane	263-353	1.75 ± 0.56	1.75 ± 0.56	~ 0	This work
		273, 298	0.439	2.7 [b]	542 [b]	Estimate, Burkholder et al. (2020)
	2-chlorobutane	268-363	2.42 ± 0.16	0.527 ± 0.123	- 463 ± 143	Kar and Rajakumar (2025)
		298	2.45 ± 0.30	*n.d.*	*n.d.*	Loison et al. (1998)
	2-bromobutane	*n.a.*	*n.a.*	*n.a.*	*n.a.*	
	2-iodobutane	*n.a.*	*n.a.*	*n.a.*	*n.a.*	

[a] *n.a.* refers to "not available" and *n.d.* refers to "not determined"; [b] see text.

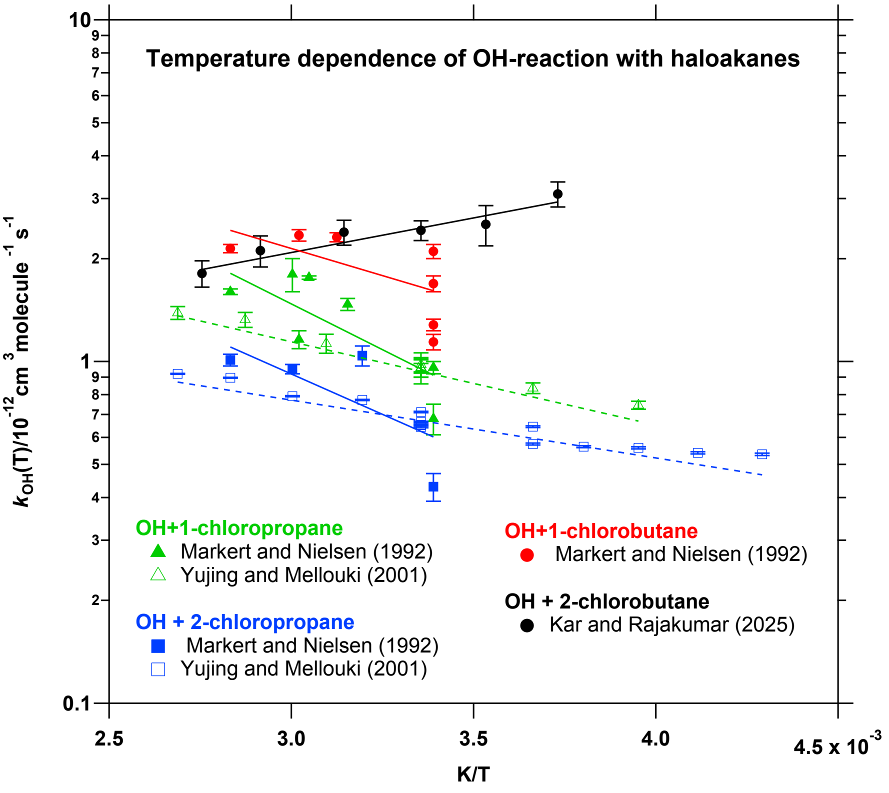

Figure S7. Comparison of the temperature dependence of k_{OH}(T) for 1-halopropanes and 2-halobutanes.

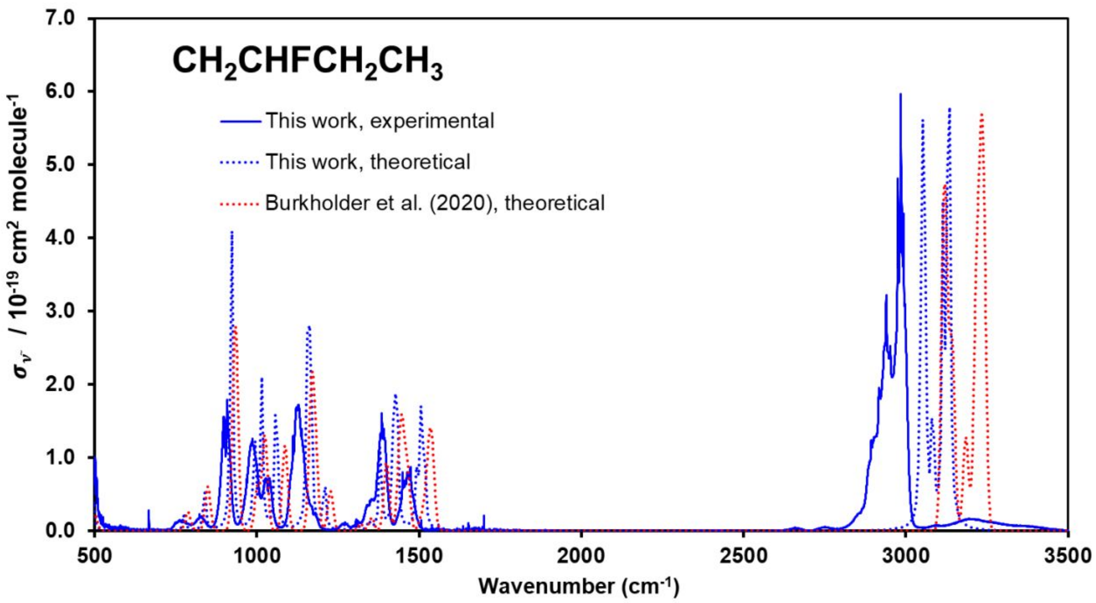

Figure S8. Infrared absorption cross sections (in base *e*) between 500 and 3,500 cm^{-1} for 2-fluorobutane at 298 K.

REFERENCES

(1) Markert, F.; Nielsen, O. J. The Reactions of OH Radicals with Chloroalkanes in the Temperature Range 295-360K. *Chem Phys Lett* 1992, *194*, 123–127. https://doi.org/10.1016/0009-2614(92)85753-W.

(2) Kar, B.; Rajakumar, B. Atmospheric Reactions of Substituted Butanes with OH Radicals: Kinetics and Atmospheric Implications. *Physical Chemistry Chemical Physics* 2025. https://doi.org/10.1039/d5cp00961h.

(3) Burkholder, J. B.; Sander, S. P.; Abbatt, J.; Barker, J. R.; Cappa, C.; Crounse, J. D.; Dibble, T. S.; Huie, R. E.; Kolb, C. E.; Kurylo, M. J.; Orkin, V. L.; Percival, C. J.; Wilmouth, D. M.; Wine, P. H. *Chemical Kinetics and Photochemical Data for Use in Atmospheric Studies, Evaluation No. 19, JPL Publication 19-5*; Pasadena, CA, 2019. http://jpldataeval.jpl.nasa.gov.

(4) Atkinson, R.; Baulch, D. L.; Cox, R. A.; Crowley, J. N.; Hampson, R. F.; Hynes, R. G.; Jenkin, M. E.; Rossi, M. J.; Troe, J. Evaluated Kinetic and Photochemical Data for Atmospheric Chemistry: Volume II-Gas Phase Reactions of Organic Species. *Atmos Chem Phys* 2006, *6*, 3625–4055. https://doi.org/10.5194/acp-6-3625-2006.

(5) Yujing, M.; Mellouki, A. Rate Constants for the Reactions of OH with Chlorinated Propanes. *Physical Chemistry Chemical Physics* 2001, *3*, 2614–2617. https://doi.org/10.1039/b102971c.

(6) Donaghy, T.; Shanahan, I.; Hande, M.; Fitzpatrick, S. Rate Constants and Atmospheric Lifetimes for the Reactions of OH Radicals and Cl Atoms with Haloalkanes. *Int J Chem Kinet* 1993, *25*, 273–284. https://doi.org/10.1002/kin.550250407.

(7) *IUPAC Task Group on Atmospheric Chemical Kinetic Data Evaluation*. https://iupac.aeris-data.fr/.

(8) Mellouki, A.; Ammann, M.; Cox, R. A.; Crowley, J. N.; Herrmann, H.; Jenkin, M. E.; McNeill, V. F.; Troe, J.; Wallington, T. J. Evaluated Kinetic and Photochemical Data for Atmospheric Chemistry: Volume VIII-Gas-Phase Reactions of Organic Species with Four, or More, Carbon Atoms (≥ C4). *Atmos Chem Phys* 2021, *21*, 4797–4808. https://doi.org/10.5194/acp-21-4797-2021.

(9) Loison, J. C.; Ley, L.; Lesclaux, R. Kinetic Study of OH Radical Reactions with Chlorobutane Isomers at 298K. *Chem Phys Lett* 1998, *296*, 350–356. https://doi.org/10.1016/S0009-2614(98)01058-6.

(10) Burkholder, J. B.; Marshall, P.; Bera, P. P.; Francisco, J. S.; Lee, T. J. Climate Metrics for C1–C4 Hydrofluorocarbons (HFCs). *J Phys Chem A* 2020, *124*, 4793–4800. https://doi.org/10.1021/acs.jpca.0c02679.
